# Concurrent Helminthic Infection Protects Schoolchildren with *Plasmodium vivax* from Anemia

**DOI:** 10.1371/journal.pone.0011206

**Published:** 2010-06-21

**Authors:** Gisely Cardoso Melo, Roberto Carlos Reyes-Lecca, Sheila Vitor-Silva, Wuelton Marcelo Monteiro, Marilaine Martins, Silvana Gomes Benzecry, Maria das Graças Costa Alecrim, Marcus Vinícius Guimarães Lacerda

**Affiliations:** 1 Tropical Medicine Foundation of Amazonas, Manaus, Amazonas, Brazil; 2 University of Brasilia, Distrito Federal, Brazil; 3 University of the State of Amazonas, Manaus, Amazonas, Brazil; 4 Nilton Lins Universitary Center, Manaus, Amazonas, Brazil; BMSI-A*STAR, Singapore

## Abstract

**Background:**

*Plasmodium vivax* is responsible for a significant portion of malaria cases worldwide, especially in Asia and Latin America, where geo-helminthiasis have a high prevalence. Impact of the interaction between vivax malaria and intestinal helminthes has been poorly explored. The objective of this study was to evaluate the influence of intestinal helminthiasis on the concentration of hemoglobin in children with *Plasmodium vivax* malaria in rural areas in the municipality of Careiro, in the Western Brazilian Amazon.

**Methodology/Principal Findings:**

A cohort study was conducted from April to November 2008, enrolling children from 5 to 14 years old in two rural areas endemic for malaria. A cross-sectional evaluation was performed in April to actively detect cases of malaria and document baseline hemoglobin and nutritional status. Children were followed-up for six months through passive case detection of malaria based on light microscopy. Throughout the follow-up interval, hemoglobin value and stool examination (three samples on alternate days) were performed on children who developed *P. vivax* malaria. For 54 schoolchildren with a single infection by *P. vivax*, hemoglobin during the malaria episode was similar to the baseline hemoglobin for children co-infected with *Ascaris lumbricoides* (n = 18), hookworm (n = 11) and *Trichuris trichiura* (n = 9). In children without intestinal helminthes, a significant decrease in the hemoglobin during the malarial attack was seen as compared to the baseline concentration. In the survival analysis, no difference was seen in the time (in days) from the baseline cross-sectional to the first malarial infection, between parasitized and non-parasitized children.

**Conclusion/Significance:**

For the first time, a cohort study showed that intestinal helminthes protect against hemoglobin decrease during an acute malarial attack by *P. vivax*.

## Introduction

Malaria is one of the most important public health problems in the world, with 3.3 billion people at risk of contracting the disease and almost one million deaths annually, mainly in children under five years [1]. In Latin America, from 2000 to 2007, 7,554,993 cases of malaria were recorded, of which 5,507,167 (72.9%) were caused by *Plasmodium vivax*. In this same period, 3,833,477 cases were reported in Brazil, mainly in the Amazon Region, with the same predominance of *P. vivax* (76.7%) [2].

Malaria contributes to hemoglobin concentration decrease through a number of mechanisms, primarily through destruction and removal of parasitized erythrocytes, and a decrease in the average life span and rate of production of red blood cells [3]. In acute cases hemolysis is frequently seen, while in chronic or repeated infections dyserythropoiesis plays an important role in the pathogenesis of anemia [4]. Few studies are available focusing in anemia and vivax malaria in Latin America [5], [6].

In Brazil, especially in the Amazon region, geo-helminthiasis have a high prevalence [7], [8]. The most common intestinal helminthes infecting people are *Ascaris lumbricoides*, hookworm and *Trichuris trichiura*. These are widely distributed in tropical countries, infecting 1.3, 1.4 and 1 billion people, respectively [9]. Hookworm are causative agents of anemia in humans [10], [11], and infections of moderate or high intensity by *Trichuris trichiura* [11], [12], [13] are also associated with anemia. Infection by *Ascaris lumbricoides* influences the nutritional status [14], but its impact on anemia is unclear.

In the Amazon region, as in many other parts of the world, endemic areas for malaria coincide with locations of high prevalence of intestinal helminthiasis [15], [16]. Recently studies have focused on the interactions between malaria and helminthiasis co-infection. Preliminary data suggest a decrease in the severity of malaria due to *P. falciparum* among those co-infected with intestinal helminthes [17].

Research on the interaction between these parasites is predominantly focused on *P. falciparum* [18], [19], [20], [21], the predominant species in Africa. However, *P. vivax* is responsible for a significant portion of malaria cases worldwide, especially in Asia and Latin America [22], and the interaction between this species and intestinal helminthes has been poorly explored.

The objective of this study was to evaluate the influence of intestinal helminthiasis on the hemoglobin concentration in children with *P. vivax* malaria in rural areas highly endemic for malaria, in the Western Brazilian Amazon.

## Methods

### Ethics Statement

The study was approved by the Ethical Review Board of the Tropical Medicine Foundation of Amazonas (approval number 1899). Parents' participants were instructed about the objectives of the study and signed an informed consent. Patients diagnosed with intestinal parasites and malaria were treated according to the guidelines of this institution.

### Area of Study

A cohort study was carried out in two schools located in two recently colonized areas devoted to agriculture (Panelão and Céu Azul Communities), from April to November 2008. These settlements are located in the Municipality of Careiro, Amazonas State. The municipality has an area of 6,124.30 km^2^ and 31,063 inhabitants. The climate is tropical and humid, with rainfall ranging from 2,100 to 2,400mm *per annum*. The municipality is connected with the capital of the state, Manaus, through a federal road (112 km of distance).

The total population of both communities is 790 persons, according to the census performed before the beginning of the study. The major economical activities are family farming, hunting and fishing. Water for drinking comes from rain water reservoirs or creeks. Garbage collection and sanitation are absent. Two health agents in each community are responsible for health care.

The studied population was composed of 236 schoolchildren 5 to 14 years old.

### Study Design

In April 2008, a cross-sectional study was conducted to actively detect malaria cases or asymptomatic Plasmodium carriers, establish baseline hemoglobin, assess nutritional status. Children who had a positive thick blood smear were treated according to the Brazilian Anti-Malarial Treatment Guidelines and not included in the analysis afterwards. Children were followed from April to November 2008, and when presented with measured or reported history of fever, a thick blood smear was performed (passive case detection). In this period, children who developed the first episode of *P. vivax* infection had a stool examination and hemoglobin concentration performed on the day of the diagnosis. After the first malarial episode, the child was not subsequently followed. Children diagnosed with *P. falciparum* or mixed infection (*P. vivax/P. falciparum*), or those who had received anti-helminthic drugs during the study interval, those who could not collect three samples of feces on alternate days, or those who declined to participate in the study were excluded from the analysis. To eliminate potential confounding factors, patients with known hemoglobinopathies, glucose 6-phosphate dehydrogenase deficiency, and/or other chronic diseases were excluded.

### Diagnosis of Malaria and Parasitemia Quantitation

Thick blood smear was prepared as recommended by the Walker technique [23] and evaluated by a local microscopist. The slides were sent to Manaus and reviewed by an experienced microscopist, who confirmed the species and determined the peripheral parasitemia, quantifying the asexual forms per 100 leukocytes counted in high-magnification fields, using an estimate of 5,000 leukocytes/mm^3^ for each child. Parasitemia was quantified as asexual parasites/mm^3^.

### Nutritional Status Assessment

Weight and height were obtained by internationally recommended methods, as follows. Weight was measured by use of a digital scale and height was assessed with the help of a tape. Body Mass Index (BMI) was calculated using the program EPI-INFO 3.4.3. BMI Z-score <−2 was defined as malnutrition, scores between −2 and Z<−1 as the risk zone; Z score between −1 and 2 normal weight and Z score >2 as obesity [24].

### Stool Examination

The search for hookworm, *Ascaris lumbricoides* and *Trichuris trichiura* was performed by examination of three samples of stool from each child, collected on alternate days. A single researcher performed all the exams, in order to avoid examiner's bias. The stool samples were stored in flasks containing 10% formalin as preservative. Flasks were labeled with the patient's name, date of collection and kept at room temperature until the end of the month, when all the stool samples were examined. Spontaneous sedimentation [25] and centrifugal-flotation in zinc sulphate solution [26] methods were applied before the samples were analyzed by direct observation with a microscope.

### Hemoglobin Concentration

Hemoglobin concentration was measured in venous blood obtained from digital puncture, using a portable HemoCue® photometer (Anglholm, Sweden).

### Statistical Analyses

Data were analyzed using SPSS® version 16.0 for Windows (SPSS Inc.® Chicago, IL, USA). Normal distribution of data was evaluated with the Kolmogorov-Smirnov test. Chi-square or Fisher's test was used to test differences in proportions, and Student t test was used to test differences in means. Non-parametric Spearman's test was used for the correlation analyses. A Kaplan-Meier survival analysis was performed in order to detect differences in the time elapsed from the baseline cross-sectional to the first malarial episode between children with and without intestinal helminthes. Log-rank test was used to test differences. Statistical significance was considered if p<0.05.

## Results

**Figure 1:**
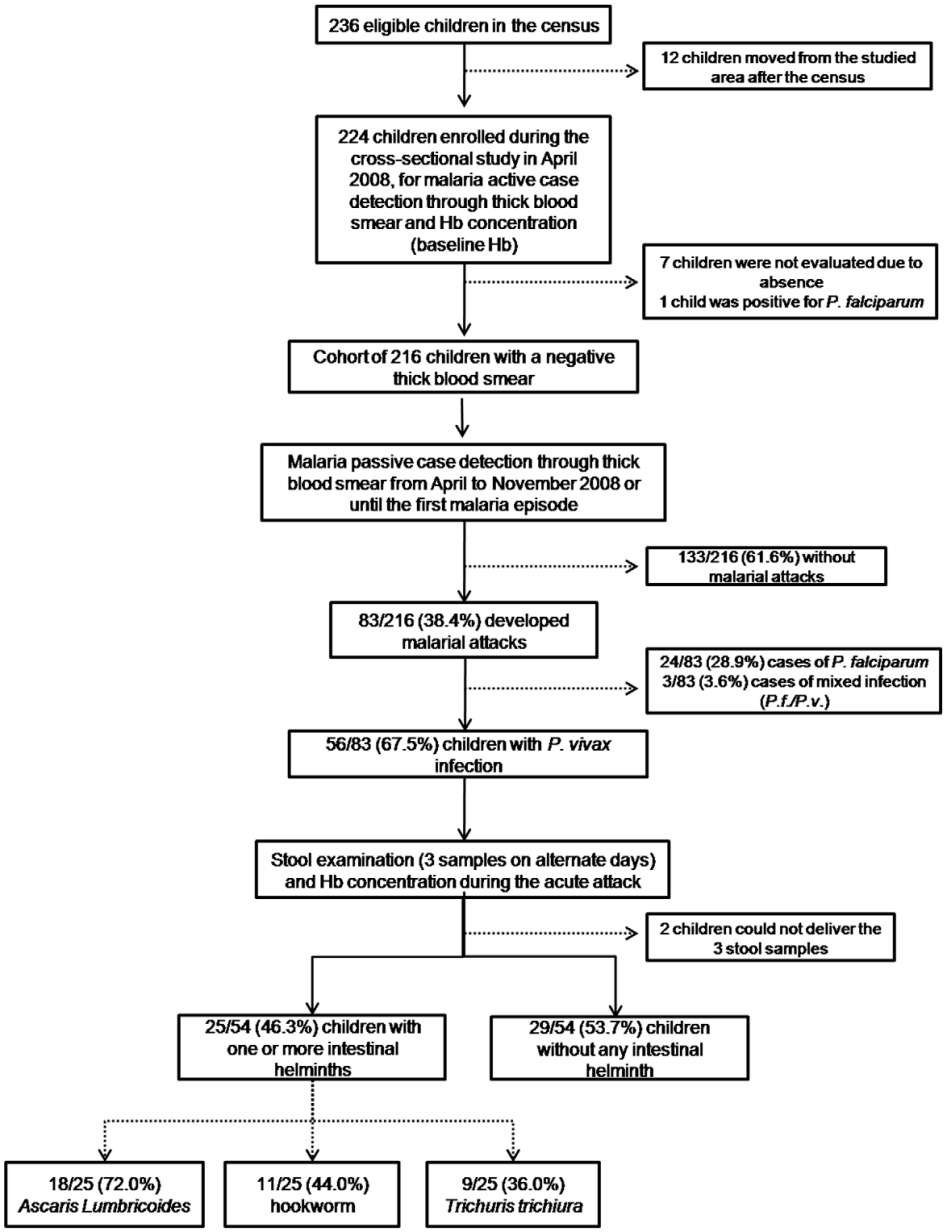
Algorithm of the study, describing the details of eligible, enrolled and analyzed children.

During the six-month follow-up interval, from 236 eligible children, 216 were enrolled in the cohort, as seen in the algorithm in Figure 1.

In Table 1, it is shown that no significant differences were observed in sex, age, nutritional status, baseline hemoglobin and/or parasitemia between the groups with and without each helminth. Children parasitized with hookworm and *Trichuris trichiura* had mothers with a significantly higher level of education as compared to the non-parasitized.

**Table 1:** Baseline characteristics of 54 schoolchildren followed from April to November 2008, in an endemic area for malaria (municipality of Careiro, Amazonas, Brazil), according to the helminth detected at stool examination during *P. vivax*.

		Ascaris lumbricoides		p	Hookworm		p	Trichuris trichiura		p
		yesn (%)	non (%)		yesn (%)	non (%)		yesn (%)	non (%)	
Sex	(n = 54)									
Male		12 (66.7)	14 (38.9)	0.05	5 (45.5)	21 (48.8)	0.84	4 (44.4)	22 (48.9)	1.00
Female		6 (33.3)	22 (61.1)		6 (54.5)	22 (51.2)		5 (55.6)	23 (51.1)	
Age (years)	(n = 54)									
5–11		16 (88.9)	26 (72.2)	0.30	8 (72.7)	34 (79.1)	0.70	8 (88.9)	34 (75.6)	0.67
12–14		2 (11.1)	10 (27.8)		3 (27.3)	9 (20.9)		1 (11.1)	11 (24.4)	
Nutritional evaluation	(n = 54)									
Nutritional risk or malnutrition		3 (16.7)	5 (14.7)	1.00	1 (10.0)	7 (16.7)	1.00	0 (0.0)	8 (17.8)	0.38
Eutrophic		15 (83.3)	31 (85.3)		10 (90.0)	36 (83.3)		9 (100.0)	37 (82.2)	
Mean of baseline Hb	(n = 54)	11.5	12,1	0.12	11.7	12.0	0.52	11.9	11.9	0.97
Mean of asexual malarial parasites/mm3	(n = 43)	2450.0	2740.4	0.76	1805.0	2874.2	0.33	2137.5	2737.1	0.61
Total		18 (100.0)	36 (100.0)		11 (100.0)	43 (100.0)		9 (100.0)	45 (100.0)	

**Figure 2:**
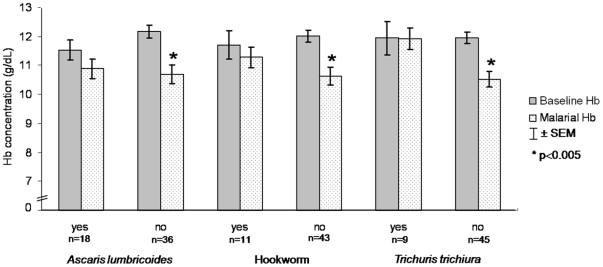
Mean (± standard error of the mean) hemoglobin concentration before (baseline Hb) and during (malarial Hb) the first *P. vivax* acute attack in 54 schoolchildren followed from April to November 2008, in an endemic area for malaria (Municipality of Careiro, Amazonas, Brazil), according to the helminth detected at stool examination.

Figure 2 shows no significant difference between the mean of baseline hemoglobin and the mean of hemoglobin during the acute *P. vivax* attack in children co-infected with *Ascaris lumbricoides* (p = 0.221), hookworm (p = 0.353) and *Trichuris trichiura* (p = 0.976). For schoolchildren with *P. vivax* infection without these helminthes at stool examination, the mean of hemoglobin during the acute malarial attack was significantly lower than the mean of the baseline hemoglobin for all the helminths (p<0.005).

**Figure 3:**
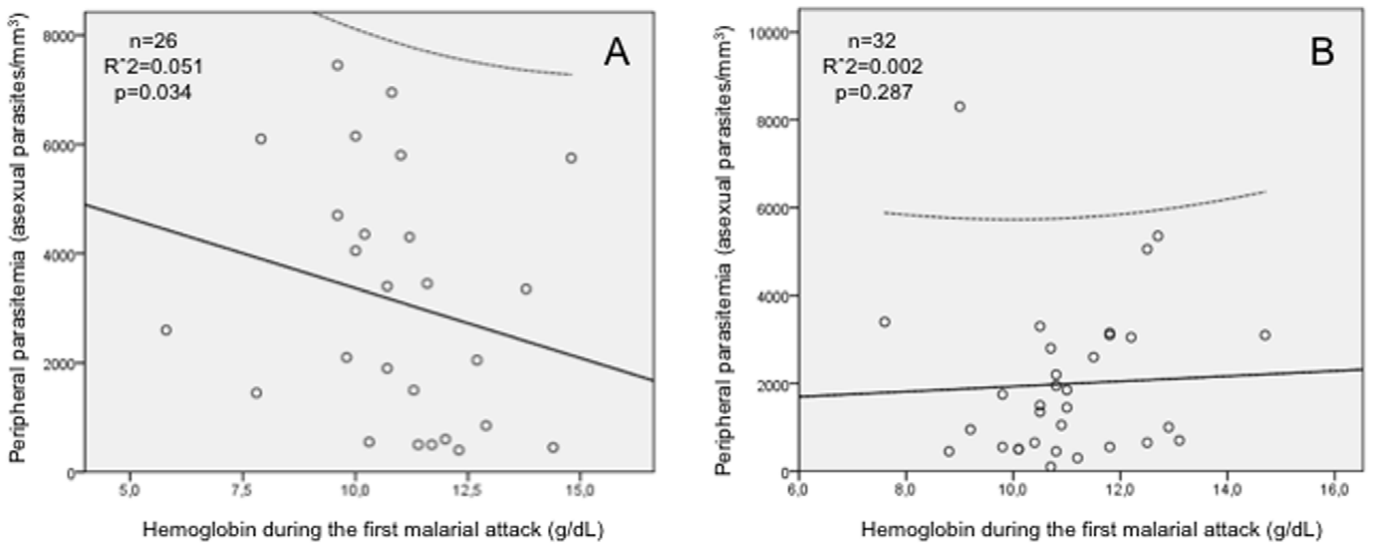
Correlation between parasitemia and hemoglobin. Correlation between parasitemia and hemoglobin during the first malarial attack in children without helminthes (A) and with helminthes (B).

Figure 3 evidences that only in the group of children without helminthes there was a significant correlation between parasitemia and the hemoglobin during the malarial attack (p = 0.034). In the co-infected group the correlation was lost (p = 0.287).

**Figure 4:**
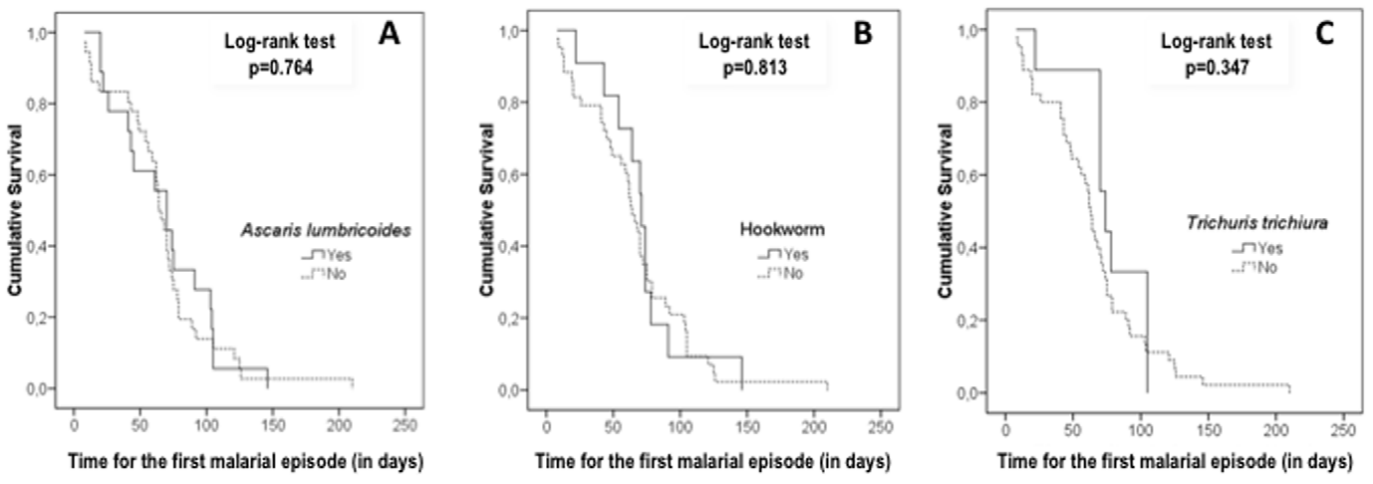
Survival analysis of the time for the first malarial episode. Kaplan-Meier survival analysis showing the time elapsed from the baseline cross-sectional to the first malarial infection (in days) in 54 schoolchildren followed from April to November 2008, with and without *Ascaris lumbricoides* (A), hookworm (B) and *Trichuris trichiura* (C).

Figure 4 evidences that there was no difference in the time elapsed from the baseline cross-sectional evaluation until the first malarial infection between the groups of parasitized and non-parasitized children.

No child required hospitalization in the local hospital or died during the course of the study, and no iron supplementation was prescribed.

## Discussion

Anemia is widely distributed and often found in developing countries [27]. The consequences of anemia are particularly severe for children and pregnant women [28], [29]. Chronic anemia during childhood is associated with retardation in physical development, cognition and school performance [30], while severe anemia (hemoglobin <5g/dL) is responsible for more than half of the deaths attributed to malaria in children under five years of age [31].

Although the etiology of anemia is complex and multifactorial, parasitic diseases, including malaria and intestinal parasites are recognized as the major cause of anemia in endemic countries [32], [33]. There are few studies evaluating the association between malaria and helminth infections as a cause of anemia in children [32], [34]. Although the association between helminthiasis and malaria is well documented in endemic regions such as Africa and Asia [17], [18], [19], [35], [36], [37], this information is not available for Latin America, where *P. vivax* predominates and malnutrition is not as frequent and severe as on the African Continent.

This study demonstrated that intestinal helminthiasis is associated with protection against a decline in hemoglobin concentration during episodes of *P. vivax* malaria. Previous studies have shown that helminth infections provide protection against infection by *P. falciparum* [17], [20], [21], [38], [39]. The first studies on this co-infection in the 1970s suggested that infection by *A. lumbricoides* was associated with biological suppression of malaria [40], [41]. More recent studies have shown that *A. lumbricoides* promotes a protective effect against cerebral malaria [17] and renal failure [42]. In Thailand, it was observed that hookworm-infected patients with mild *P. falciparum* malaria had lower mean of admission temperatures than patients without hookworm [38]. In our children, temperature was not measured because most of the population was already using antipyretics when they presented to the local health post. Using clinical severity in general as an endpoint of protection conferred by helminths in the case of *P. vivax* infection would not be appropriate in our field study, in an unstable transmission area, where no child needed hospitalization or died during the course of the study. Further studies are needed focusing in patients from tertiary care centers, where clinical severity is more common [43], [44].

The influence of helminthic infections on malaria is not entirely straightforward. In Madagascar, it was observed that infection by *A. lumbricoides* is associated with lower parasitemia in *P. falciparum* malaria [20], [21]. However, in Senegal, the rate of malarial attack was higher among children co-infected with *S. mansoni* [45] and an association between *A. lumbricoides* infection and severe malaria was found elsewhere, despite of the loose definition of severity in this particular case [46]. In Thailand, it was observed that helminth infections are associated with increased incidence of *P. falciparum* [18]. In our study, the degree of asexual parasitemia by *P. vivax* was not different between children with and without helminthiasis. This is probably because they were diagnosed very early (diagnostic tests are available for everyone at no cost in the community) and also because *P. vivax* lacks the development of high parasitemia due to its selective reticulocyte invasion. In children with severe falciparum malaria, co-infection with helminthes leaded to the decrease in circulating reticulocyte counts [47]. Therefore, helminthes may also influence *P. vivax*-triggered anemia through the reduction of reticulocytes, although this issue was not addressed in our study. The presence of helminthes contributed to a lack of association between parasitemia and hemoglobin in this group, suggesting that helminthes may modulate the impact of parasitemia upon anemia. Recently, it was shown that the frequency of CD4+CD25+FoxP3+ regulatory T cells correlates with *P. vivax* parasitemia [48]. However, another study has shown that the presence of helminthes associates with a suppressive Treg activity *in vitro* for *P. falciparum* [49]. Further studies are needed to confirm this speculation. Children with *P. falciparum* infection or mixed infection (*P. vivax/P. falciparum*) were not analyzed as a comparator group due to the small number of cases. An active case detection design (using thick blood smear and PCR, and children without malaria as a control group) could additionally identify if intestinal helminths are able to confer protection against the clinical manifestations of the disease and/or the state of asymptomatic carrier of the parasite.

Despite evidence that malaria/intestinal helminthiasis co-infection increases the risk of anemia, little is known about the impact of co-infection on anemia amongst different age groups [32]. In preschool, co-infection was associated with a lower hemoglobin concentration as compared to children without malaria [50]. This control group (children without malaria) was not evaluated in our study. Among schoolchildren, hemoglobin was lower in the co-infected as opposed to children with only one infection [51]. However, no significant difference was observed among pregnant women, although single infections by malaria or hookworm had an impact on the concentration of hemoglobin [52]. It is important to emphasize that all these studies were performed using a cross-sectional study design. This poses some difficulty in the comparison with our data. The likelihood that these differences may be distinct in each age group is also very high, because anemia due to *P. vivax* infection is mostly seen in children under 5 years of age [44]. Children this young were not enrolled in our study.

Surprisingly, not even hookworm infection was associated with a decrease in the hemoglobin concentration in children with malaria. Due to the anemic effect of this helminth *per se*, we expected that in this group anemia would be more severe. However, it is important to note that in all the groups children sometimes had more than one helminth at stool examination, precluding an isolated analysis. Due to the lack of a professional who could perform the Kato-Katz technique in the field to quantify the intestinal helminth load in fresh stool, we could not evaluate for the potential effects of intensity of helminth infection. Further studies should explore this aspect.

A clear bias of this study could have been the longer time elapsed from the baseline cross-sectional until the first malarial episode in children without intestinal helminthes, subjected therefore to other determinants of anemia. However, for every studied helminth, this time was very similar, what additionally suggests that intestinal helminthes do not contribute to *P. vivax* clinical protection.

It was noted that helminthes protect against the reduction of hemoglobin during acute malarial attacks by *P. vivax*. To our knowledge, this is the first report to demonstrate the impact of helminthic co-infection on a complication triggered by *P. vivax*, based on a cohort study. The mechanism by which helminthes apparently protect against a decrease in hemoglobin in vivax malaria is unknown. One speculates that high levels of the Th2 cytokines (IL-10) during helminth infection counteract the Th1 cytokines (TNF) induced by malaria to prevent the development of severe anemia [4]. The overall Th2/Th1 balance, the homeostatic role of interleukin 10 and TGF-β as modulators of the immune response [53], and the role of the CD23/NO pathway in reducing sequestration [17] are additional possible mechanisms of protection against severe malaria. Other epidemiological studies are needed to confirm our findings with a higher number of patients, and especially in preschool children where the effect could be more evident. Studies are also necessary to evaluate the effect of the intensity of helminth load on the course of *P. vivax* infection.
